# Can Transcranial Direct-Current Stimulation Alone or Combined With Cognitive Training Be Used as a Clinical Intervention to Improve Cognitive Functioning in Persons With Mild Cognitive Impairment and Dementia? A Systematic Review and Meta-Analysis

**DOI:** 10.3389/fnhum.2018.00416

**Published:** 2018-10-16

**Authors:** Pablo Cruz Gonzalez, Kenneth N. K. Fong, Raymond C. K. Chung, Kin-Hung Ting, Lawla L. F. Law, Ted Brown

**Affiliations:** ^1^Department of Rehabilitation Sciences, The Hong Kong Polytechnic University, Kowloon, Hong Kong; ^2^University Research Facility in Behavioral and Systems Neuroscience, The Hong Kong Polytechnic University, Kowloon, Hong Kong; ^3^School of Medical and Health Sciences, Tung Wah College, Kowloon, Hong Kong; ^4^Department of Occupational Therapy, Monash University, Melbourne, VIC, Australia

**Keywords:** tDCS (transcranial direct-current stimulation), neuromodulation, MCI (mild cognitive impairment), dementia, cognitive rehabilitation, cognitive training, systematic review, meta-analysis

## Abstract

**Background:** Transcranial direct-current stimulation (tDCS) facilitates cognitive improvement in healthy and pathological populations. It has been increasingly used in cases of mild cognitive impairment (MCI) and dementia. Our research question is: Can tDCS serve as a clinical intervention for improving the cognitive functions of persons with MCI (PwMCI) and dementia (PwD)?

**Objective:** This systematic review evaluated the evidence to determine the efficacy of tDCS in improving cognitive outcomes in PwD and PwMCI.

**Methods:** A systematic review was conducted of studies published up to November 2017 involving tDCS in cases of MCI and dementia. Studies were ranked according to the level of evidence (Oxford Center for Evidence-Based Medicine) and assessed for methodological quality (Risk of Bias Tool in the Cochrane Handbook for Systematic Reviews of Interventions). Data was extracted on all protocol variables to establish a reference framework for clinical interventions. Different modalities, tDCS alone or combined with cognitive training, compared with sham tDCS were examined in both short and long-term effects. Four randomized control trials (RCTs) with memory outcomes were pooled using the fixed-effect model for the meta-analysis.

**Results:** Twelve studies with 195 PwD and four with 53 PwMCI met the inclusion criteria. Eleven articles were ranked as Level 1b. The results on the meta-analysis on pooled effects of memory indicated a statistically significant medium effect size of 0.39 (*p* = 0.04) for immediate effects. This improvement was not maintained in the long term 0.15 (*p* = 0.44).

**Conclusion:** tDCS improves memory in PwD in the short term, it also seems to have a mild positive effect on memory and language in PwMCI. However, there is no conclusive advantage in coupling tDCS with cognitive training. More rigorous evidence is needed to establish whether tDCS can serve as an evidence-based intervention for both populations.

## Introduction

Transcranial direct-current stimulation (tDCS) is a type of non-invasive brain stimulation (NIBS). tDCS delivers weak direct currents to the brain that can alter spontaneous firing rates on neural activity, which subsequently translates into behavioral changes (Nitsche et al., 2008). It is a process that has been described as “portable, painless, inexpensive and safe” (Kadosh et al., 2012). During the administration of tDCS, depolarization or hyperpolarization of the neuronal membrane of target neurons may be induced, even though the small electric fields of tDCS are considered to be below the intensity required to evoke action potentials (Nitsche et al., 2003; Miniussi et al., 2013; Tatti et al., 2016). In other words, tDCS causes a shift in the membrane potential threshold which is likely to change the probability that an incoming action potential will result in post-synaptic firing during and after its administration (Prehn and Flöel, 2015). Such changes in neuronal excitability modulates the cognitive processes and tDCS can induce physiological processes. Due to the proposed resemblance of the effects of tDCS and cognitive processes on cerebral physiology, researchers have been using NIBS to alter cognition (Kuo and Nitsche, 2012; Prehn and Flöel, 2015).

Mild Cognitive Impairment (MCI) is defined as the stage between normal and dementia-type pathological aging. MCI is a syndrome of cognitive decline in non-demented persons that does not affect the capacity to be independent in activities of daily living (ADLs; Portet et al., 2006). In contrast, people who suffer from dementia present a more severe cognitive decline and do not preserve independence in functional abilities and ADLs (Langa and Levine, 2014). Epidemiological investigations suggest a range of prevalence for MCI of 7–24% among adults aged over 65, and the manifestation of MCI is consistently shown to have a high risk of progression to dementia (Langa and Levine, 2014; Petersen et al., 2014). To date, there is no pharmaceutical treatment shown to be effective in improving cognitive functioning in MCI and dementia (Langa and Levine, 2014), although cognitive training interventions show promise for improving targeted cognitive functions in elderly persons without cognitive impairments (Ball et al., 2002). Cognitive Rehabilitation (CR) is defined as “the therapeutic process of increasing or improving an individual's capacity to process and use incoming information so as to allow increased functioning in everyday life.” This includes methods to train and restore cognitive functioning as well as compensatory techniques (Sohlberg and Mateer, 1989, p. 871).

CR is therefore essential and research has indicated that NIBS can positively affect the cognitive performance of populations affected by cognitive disorders (Miniussi et al., 2008). Differences in tDCS experimental protocols regarding the parameters employed such as the montage, the current, the intensity or the size of the electrodes can affect the electric field strength. All of these variables contribute to increase the heterogeneity of the electric field's properties among studies thus producing different outcomes (Woods et al., 2016). Furthermore, targeting a neural network with tDCS while it is engaged by a cognitive stimulation activity, during or after the administration of tDCS, may yield better therapeutic effects than stimulating the same cortical region lacking cognitive stimuli (Cruz Gonzalez et al., 2018). tDCS may increase the strength of transmission across synaptic circuits in pathways that are stimulated by cognitive practice. Thus, coupling both techniques could create a synergistic positive effect on behavior (Miniussi et al., 2013; Birba et al., 2017; Cruz Gonzalez et al., 2018). The effectiveness of tDCS in CR targeting people with MCI or dementia must therefore be established. It is fundamentally important to learn about all the different configurations and protocols in which tDCS has been employed to assess its utility.

We systematically reviewed the literature regarding effects of tDCS on persons with MCI and dementia to address the following questions: (1) Does tDCS alone improve cognitive functioning in persons with MCI and dementia? (2) Does tDCS coupled with cognitive training, or as a priming to other cognitive interventions yield greater benefits in cognitive functioning than the administration of tDCS alone? (3) Are the effects of tDCS on the cognitive functions able to maintain across time?

In this study, we reviewed and evaluated the effects of tDCS on cognitive functions in people with MCI or dementia from all the available clinical trials. A systematic review of the available information up to the present will enable researchers to better understand the potential of tDCS to offer solutions for cognitive deterioration, with the aim of outlining more robust interventions in the future for people with MCI and dementia. Other reviews involving the use of different NIBS on healthy aging (Prehn and Flöel, 2015), dementia (Freitas et al., 2011; Hsu et al., 2015), MCI (Birba et al., 2017) have been carried out since 2011, but we provide an update and meta-analysis of recent trials to focus exclusively on the use of tDCS in MCI and dementia populations.

## Methods

### Eligibility criteria

We performed a systematic review and meta-analysis following the PRISMA guidelines (Liberati et al., 2009). Studies were selected based on the following criteria:

- Participants: Participants included in the study were older adults with MCI and persons with a diagnosis of dementia. The criteria for MCI includes (a) subjective memory complaint; (b) objective cognitive decline; (c) preserved ADLs, and (d) not demented (Petersen et al., 1999). The diagnosis of dementia followed the criteria of the NINCDS-ADRDA (McKhann et al., 1984) and the DSM-IV (American Psychiatric Association, 2000). Participants with any other neurological disease that was not dementia, such as only the Parkinson's type, were excluded.
- Interventions: tDCS alone (anodal, cathodal, or sham), or a combination of tDCS (online or offline) with an additional cognitive task (CT).
- Comparisons: The comparison group could be a placebo with sham tDCS, sham tDCS in combination with a CT, or a control group performing a cognitive intervention. In order to establish evidence on tDCS protocols for people with MCI or dementia, studies without sham tDCS were included.
- Outcome measurements: The outcomes were measurements of cognitive functions and neuroimaging techniques.
- Study design: All clinical trials published in English from January 2007 to November 2017 were included.

### Search strategy

Studies were identified by a systematic literature search in the following databases: PubMed, Web of Science, Science Direct, MEDLINE, and PsycINFO. A search was performed combining all the chosen keywords across the above databases. The keywords and the search strategy are presented in Table 1. A hand search was also performed to identify relevant studies.

**Table 1:** Sample search strategy and databases.

**Search strategy**	**Database**	**Articles yielded**
Aged OR aging OR old adult OR old people OR old person OR aged OR aging/aging OR elder OR geriatric	PubMed	2282878
	Web of science	20020579
	Science direct	160098
	Medline	2215444
	PsycINFO	990595
Mild cognitive impairment OR MCI OR subtle cognitive impairment OR mild dementia OR prodromal dementia	PubMed	39043
	Web of science	32402
	Science direct	26522
	Medline	18949
	PsycINFO	13300
Dementia OR Alzheimer's disease OR AD OR vascular dementia OR VD OR dementia with Lewy bodies OR DLB OR mixed dementia OR frontotemporal dementia	PubMed	680614
	Web of science	230907
	Science direct	8365
	Medline	218682
	PsycINFO	67559
1 AND 2 OR 3	PubMed	688964
	Web of science	234611
	Science direct	1936
	Medline	221967
	PsycINFO	69699
Cognition OR executive function OR attention OR memory or working memory OR cognitive training OR cognitive intervention OR cognitive stimulation OR cognitive rehabilitation OR cognitive remediation OR brain training OR mental training OR memory training OR mnemonic training OR executive function training OR attention training or working memory training	PubMed Web of science Science direct Medline PsycINFO	688598 934342 24133 462185 815917
Transcranial direct-current stimulation OR tDCS OR direct-current stimulation OR TES OR DC stimulation OR electrical stimulation OR transcranial stimulation OR non-invasive brain stimulation OR NIBS OR neuromodulation	PubMed	65155
	Web of science	60269
	Science direct	11106
	Medline	44985
	PsycINFO	36695
4 AND 5 AND 6	PubMed	1135
	Web of science	601
	Science direct	43
	Medline	460
	PsycINFO	333
Randomized control trials OR clinical trial OR crossover studies OR case control studies OR case series OR case report OR placebos OR sham OR control	PubMed	3021385
	Web of science	3889523
	Science direct	231043
	Medline	2521985
	PsycINFO	744877
7 AND 8	PubMed	434
	Web of science	317
	Science direct	31
	Medline	235
	PsycINFO	181

### Selection criteria

After removing duplicates, the abstracts of the articles retrieved were screened to make a final decision for further review. Two investigators realized the search and the selection of studies to be included. Any disagreements were resolved by a third reviewer.

### Data extraction

The data extracted from the selected studies were conducted by two investigators using a standardized data extraction sheet which included study design, study population, number of participants, mean participant age, gender ratio, general cognitive level, number of intervention sessions, experimental/sham tDCS parameters, combination of tDCS with other interventions, outcome measures, neuroimaging techniques, assessment sequence, follow-up, effect(s) of the intervention, and intervention safety reports.

### Methodological quality

The studies selected for review were categorized and leveled according to their design based on the hierarchy level of evidence [Oxford Center for Evidence-based Medicine—Levels of Evidence (March 2009)—CEBM^1^]. All randomized control trials (RCTs) were then rated by the first two authors using the Risk of Bias Tool in the Cochrane Handbook for Systematic Reviews of Interventions (Higgins and Green, 2008).

### Data analysis

Only RCTs, excluding crossover designs, were considered for meta-analysis. In some cases, authors were contacted to obtain data from their studies. After the review of the clinical methodology's heterogeneity of each study (Table 2), the selected papers were further assessed for statistical heterogeneity, using the I-squared and Chi-squared statistics of the outcome measures.

**Table 2:** Methodology's heterogeneity assessment of RCT'S.

**Study**	**Stimulated region**	**Intensity (mA)**	**Sessions**	**Duration (min)**
André et al., 2016	LDLPFC	2	4	20
Cotelli et al., 2014	LDLPFC	2	10	25
Khedr et al., 2014	LDLPFC	2	10	25
Suemoto et al., 2014	LDLPFC	2	6	20
Bystad et al., 2016a	Temporal cortex (T3)	2	6	30

*LDPFC, Left dorsolateral prefrontal cortex*.

Data of pooled memory outcomes comparing: (1) Short-term effects of tDCS treatments vs. sham tDCS that targeted the dorsolateral prefrontal cortex (DLPFC) were calculated based on the differences between post-intervention evaluations relative to the baseline to assess the immediate effects of tDCS; (2) Long-term effects of tDCS treatments vs. sham tDCS that targeted the DLPFC; were assessed according to the differences between follow-up evaluations relative to the baseline.

All outcomes were analyzed as continuous variables with the mean change, the largest standard deviation, and the sample size in each group. The standardized mean difference and 95% confidence intervals were calculated for all meta-analyses using the fixed-effect model. The effect size was considered to be small between 0.2–0.49, moderate (0.5–0.79), and a value of 0.8 or above was considered to be large (Cohen, 1992). If *I*^2^ was below 40%, it was considered to not represent statistical heterogeneity. Otherwise, the random-effect model was used instead. Significance was set at *p* = 0.05 and both meta-analyses were conducted using Review Manager Software 5.3.

## Results

### Study selection

**Figure 1:**
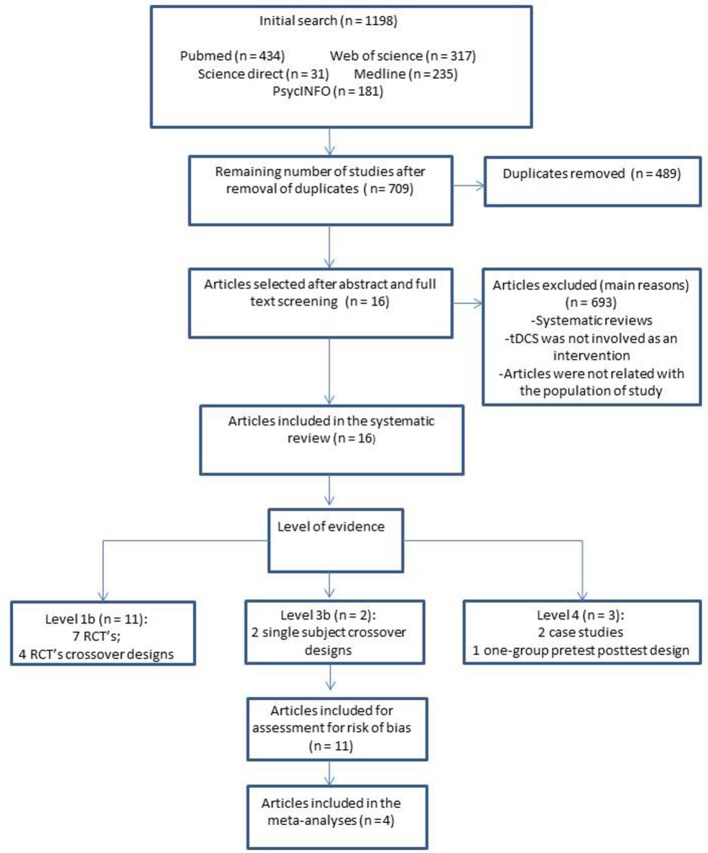
Flow chart for study selection and level of evidence. RCT's, Randomized control trials.

The search strategy identified 1,198 published articles from the selected databases: PubMed (*n* = 434), Web of Science (*n* = 317), Science Direct (*n* = 31), Medline (*n* = 235), and PsycINFO (*n* = 181) (Table 1). Sixteen articles met the eligibility criteria (Figure 1).

### Study characteristics

Eleven studies (Ferrucci et al., 2008; Boggio et al., 2009, 2012; Cotelli et al., 2014; Khedr et al., 2014; Suemoto et al., 2014; Penolazzi et al., 2015; André et al., 2016; Bystad et al., 2016a,b, 2017; Costa et al., 2017) involved the application of tDCS on persons with dementia (PwD). These articles included three randomized crossover studies (Ferrucci et al., 2008; Boggio et al., 2009, 2012), five RCTs (Cotelli et al., 2014; Khedr et al., 2014; Suemoto et al., 2014; André et al., 2016; Bystad et al., 2016a), two single-subject pretest-post-test case studies (Bystad et al., 2016b, 2017), and two single-subject crossover-design studies (Penolazzi et al., 2015; Costa et al., 2017). Four articles (Meinzer et al., 2015; Yun et al., 2016; Ladenbauer et al., 2017; Murugaraja et al., 2017) exposed persons with MCI (PwMCI) to the application of tDCS. These four studies each used a different design: a randomized crossover (Meinzer et al., 2015), an RCT (Yun et al., 2016), a group pretest-post-test (Murugaraja et al., 2017), and a balanced crossover (Ladenbauer et al., 2017).

These studies included a total of 195 participants with dementia and 53 participants with MCI. Eleven studies applied tDCS “alone” (Ferrucci et al., 2008; Boggio et al., 2012; Khedr et al., 2014; Suemoto et al., 2014; André et al., 2016; Bystad et al., 2016a,b, 2017; Yun et al., 2016; Ladenbauer et al., 2017; Murugaraja et al., 2017) and five paired tDCS with CT (Boggio et al., 2009; Cotelli et al., 2014; Meinzer et al., 2015; Penolazzi et al., 2015; Costa et al., 2017). The details of the studies' characteristics and protocols are set out in Table 3.

**Table 3:** Study characteristics.

**Study and design**	**Participants**	**tDCS montage**	**Sham tDCS montage**	**Number of sessions**	**Combination with other intervention**	**Outcomes**	**Assessment sequence**	**Effect of intervention**
**AD/MD**								
Ferrucci et al., 2008 Randomized crossover design	*N* = 10 (3 groups) AD participants MMSE = 22.7 ± 1.8 Age = 75.2 ± 7.3 70% Females	1.5 mA 15 min (1) Anode (P3-T5) Cathode (right deltoids) (2) Cathode (P6-T4) Anode (right deltoids)	10 s Same tDCS montage	1	No	Assessments: WRT (modified from ADAS-cog), VAT (exogenous cue version of the Posner paradigm) Imaging: No	At baseline, 30 min after tDCS 1 week wash out period FUP: No	Accuracy in WRT increased significantly after anodal tDCS but decreased after cathodal tDCS Safety: Itching sensation
Boggio et al., 2009 Randomized crossover design	*N* = 10 (3 groups) AD participants MMSE = 17 ± 4.9 Age = 79.1 ± 8.8 60% Females	2 mA 30 min 35 cm^2^ (1) Anode (LDPFC) Cathode (Fp2) (2) Anode (T7) Cathode (FP2)	30 s Same tDCS montage	1	VRT with faces (IBV software); Stroop test; DST (starting 10 min after the onset of the stimulation)	Assessments: VRT, Stroop test, DST Imaging: No	10 min after tDCS onset 2 days wash out period FUP: No	A significant effect of both tDCS experimental conditions on VRT, as compared with sham tDCS Safety: No adverse effects
Boggio et al., 2012 Randomized crossover design	*N* = 15 (2 groups) AD participants MMSE = 20 ± 3 Age = 79.05 ± 8.2 46.6% Females	2 mA 30 min Anode (T3 and T4) 35 cm^2^ Cathode (right deltoids) 64 cm^2^	30 s Same tDCS montage	5 (in a row)	No	Assessments: MMSE, Adas-Cog, VRT (IBV software), VAT (using endogenous cue version of the Posner task) Imaging: No	At baseline, right after the last tDCS session, 1 week and 1 month FUP Average wash out period 71.1 days	VRT improved significantly after anodal tDCS than after sham tDCS VRT performance kept improving in tDCS group at 1 month FUP Safety: No adverse effects
Cotelli et al., 2014 RCT	*N* = 36 (3 groups) AD participants MMSE; expC = 20.1 ± 2.4 expM = 22.1 ± 2.3 sham = 20.8 ± 2.1 Age; expC = 76.6 ± 4.6; expM = 78.2 ± 5.2 sham = 74.7 ± 6.1 Female proportion; expC = 83.3% expM = 83.3% sham = 75%	2 mA 25 min Anode (LDLPFC) 25 cm^2^ Cathode (right deltoids) 50 cm^2^	40 s (20 s at first, 20s at the end) Same tDCS montage	10 (5 per week for 2 weeks)	Memory training (based on the performance of the, FNAT, at the baseline) or motor training Both interventions started at the same time as the onset of tDCS	Assessments: FNAT, MMSE, ADL, IADL, Tinetti scale, NPIT, Picture naming task, BADA, RMBT, Rey auditory verbal learning test, Complex figure-copy, TMT Imaging: No	At baseline, post-intervention, 3 and 6 months FUP	tDCS plus memory training and sham tDCS plus memory training showed significantly improved performance on FNAT compared with the tDCS plus motor training group after the intervention and at 12 weeks FUP Safety: No adverse effects
Suemoto et al., 2014 RCT	*N* = 40 (2 groups) AD participants MMSE; Exp = 15.0 ± 3.1 sham = 15.4 ± 2.6 Age; Exp = 79.4 ± 7.1 sham = 81.6 ± 8.0 Female proportion; exp = 37.5% sham = 32.5%	2 mA 20 min 35 cm^2^ (1) Anode (LDLPFC) 25 cm2 Cathode (Fp2)	20 s Same tDCS montage	6 (during 2 weeks)	No	Assessments: Apathy Scale; ADAS-Cog (word list learning, word recognition and digit cancellation) Imaging: No	At baseline, post-intervention, 1 week FUP	No significant effects Safety: Tingling, sking redenss, scalp burning
Khedr et al., 2014 RCT	*N* = 34; NexpA = 11 NexpC = 12 Nsham = 11 AD participants MMSE; expA = 18.4 ± 3.9 expC = 18.8 ± 2.9 sham = 16.9 ± 2.9 Age; expA = 68.5 ± 7.2 expC = 70.7 ± 5.4 sham = 67.3 ± 5.9 Female proportion; expA = 45.4% expC = 33.3% sham = 54.5%	2 mA 25 min Anode (LDPFC) 24 cm^2^ Cathode (Fp2) 100 cm^2^ (2) Cathode (LDPFC) 24 cm2 Anode (Fp2) 100 cm2	40 s (20 s at first, 20s at the end) Same tDCS montage	10 (in a row)	No	Assessments: MMSE, WAIS-III Imaging: ERP, resting motor threshold, cortical silent periods	At baseline, post-intervention, 1 and 2 months FUP	WAIS IQ performance significantly improved after cathodal tDCS, MMSE improved and reduced P300 latency occurred after both anodal and cathodal tDCS Safety: Itching, headache and dizziness
Bystad et al., 2016a RCT	*N* = 25; Nexp = 12 Nsham = 13 AD participants MMSE; exp = 20.5 ± 8.0; sham = 22.1 ± 13.0 Age; exp = 70.25 ± 21.0; sham = 75.0 ± 30.0 Female proportion; exp = 42% sham = 47%	2mA 30 min 35 cm2 Anode (T3) Cathode (Fp2)	60 s (30s at first, 30s at the end) Same tDCS montage	6 (in 10 days)	No	Assessments: CVLT-II, MMSE, clock-drawing test; TMT, WAIS (Abbreviated version) Imaging: No	At baseline, post-intervention FUP: No	No significant effects Safety: No adverse effects
Bystad et al., 2016b Case study	*N* = 1 AD case MMSE = 23.2 Age = 59 0% Females	2mA 30 min 35 cm^2^ Anode (T3) Cathode (Fp2)	No sham	12 (during a 6-day period, twice a day)	No	Assessments: CVLT-II, MMSE Imaging: EEG	At baseline, 2 days after the last session, 2 months FUP EEG at baseline and 2 months FUP	Significantly improvement on MMSE. CVLT-II delayed recall test was clinically significant No changes in EEG Safety: No adverse effects
Bystad et al., 2017 Case study	*N* = 1 AD case MMSE = 20 Age = 60 0% Females	2mA 30 min 35 cm2 Anode (T3) Cathode (Fp2)	No sham	Daily (for 8 months)	No	Assessments: RBANS Imaging: NO	Baseline, at 5 months, at 8 months FUP: No	The patient's cognitive functions were stabilized except for visuospatial functioning. At 8 months, immediate recall and delayed recall improved Safety: Tingling and itchy sensation
Penolazzi et al., 2015 Single subject crossover design	*N* = 1 (2 groups) AD case MMSE = 23.2 Age = 60 0% Females	2 mA 20 min Anode (LDPFC) 35 cm2 Cathode (Fp2) 100 cm2	10 s Same tDCS montage	10 (in 2 weeks)	WRT; VWMT; PFT; CPT (All these activities were administered right after the tDCS administration for 45 min)	Assessments: WRT, VWMT, PFT, CPT, DST, TMT, overlapping figures, clock drawing Imaging: No	At baseline, post-intervention,2 weeks FUP 2 month wash out period	A significant accuracy improvement in WMT for tDCS + CT Safety: No adverse effects
Costa et al., 2017 Single subject crossover design	*N* = 1 (2 groups) AD case MMSE = 14.27 Age = 67 100% Females	2 mA 30 min 35 cm^2^ Anode (Broca's area) Cathode (Fp2)	30 s Same tDCS montage	5	Linguistic exercises; as writing-to-dictation, reading aloud, and repetition of words and pseudowords. (exercise were administered 7 min after the onset of tDCS)	Assessments: Naming, auditory, comprehension of nouns and verbs tasks Imaging: No	At baseline, immediately after end of intervention, 2 weeks FUP 2 week wash out period	Significant improvement of comprehension of verbs Safety: No adverse effects
André et al., 2016 RCT	*N* = 21; Nexp = 19 Nsham = 9 VD/MD participants MMSE; exp = 24.5 ± 1.8 sham = 22.4 ± 2.6 Age; exp = 80.3 ± 5.8 sham = 75.8 ± 7.4 Females	2 mA 20 min 35 cm^2^ Anode (LDPFC) Cathode (Fp2)	8 s Same tDCS montage	4 (in a row)	No	Assessments: ADAS, picture-naming task, 2-back task, Go/no-go task Imaging: No	Baseline, after intervention, 2 weeks FUP	2-back task and the go/no-go test improved. Picture naming task increased the number of memorized words after intervention Safety: No adverse effects
**MCI**								
Meinzer et al., 2015 Randomized crossover design	*N* = 18 (2 groups) MCI participants MMSE = 27.17 ± 1.34 Age = 67.44 ± 7.27 Females 38.8%	1 mA 20 min Anode (IFG) Cathode (Fp2)	30 s Same tDCS montage	1	Overt semantic word-retrieval task	Assessments: Overt semantic word-retrieval task Imaging: fMRI	Anodal tDCS vs. sham tDCS with concurrent fMRI recording during a word-retrieval task and resting state One week wash out period FUP: No	Significant improvement of the semantic word-retrieval task to the level of healthy controls Reduced task-related prefrontal hyperactivity during resting-state fMRI Safety: No adverse effects
Yun et al., 2016 RCT	*N* = 16 MCI participants MMSE; exp = 26.75 ± 1.58; sham = 25.12 ± 2.74 Age; exp = 74.75± 7.47 sham = 73.12 ± 4.25 Female distribution; exp = 37.5% sham = 25%	2 mA 20 min 25 cm^2^ Anode (LDPFC) Cathode (RDPFC)	20 s Same tDCS montage	9 (3 times per week for 3 weeks)	No	Assessments: Modified MMQ Imaging: PET	Baseline, post-intervention FUP: No	Subjective memory satisfaction and memory strategies significantly improved. Increased regional cerebral metabolism Safety: No adverse effects
Murugaraja et al., 2017 One group pretest-post-test	*N* = 11 MCI participants MMSE = 28 Age = 59.6 ± 4.3 Females 54.5%	2 mA 30 min 35 cm^2^ Anode (IFG) Cathode (Fp2)	No sham	5 (in a row)	No	Assessments: PMIT Imaging: No	Baseline and 1 h after end of the intervention 1 month FUP	Immediate and delayed recall performance improved, persisting at 1 month FUP Safety: Pricking, burning sensation
Ladenbauer et al., 2017 Randomized crossover design	*N* = 8 (2 groups) MCI participants MMSE = 28.3 ± 1.4 Mean age = 71 ± 9 Females 43.7 %	so-tDCS frequency of 0.75 Hz (0–262.5 uA) 5 min blocks(3–5 blocks in total) 8 mm Anodes (F3 and F4) Cathodes (mastoids)	Same tDCS montage tDCS device remained off	1	No	Assessments: Visuospatial memory task, verbal memory task, sequential finger tapping task Imaging: EEG	Cognitive test at baseline and after tDCS and EEG during tDCS. 2 weeks wash-out period. FUP: No	Visual declarative memory improved so-tDCS significantly increased overall SO and spindle power Safety: Tingling sensation

*AD, Alzheimer's disease; ADAS-cog, Alzheimer's disease assessment scale-Cog; BADA, battery for analysis of aphasic deficits; CVLT-II, California verbal learning Test; CPT, continuous performance task; DST, digit span test; expC, cognitive experimental group; exp, experimental group; expA, anodal experimental group; expC, cathodal experimental group; expM, motor experimental group; FNAT, face-name memory association task; FUP, Follow-up; I/ADL, instrumental/activities of daily living; IFG, inferior frontal gyrus; L/RDFPC, left/right dorsolateral prefrontal cortex; MD, Mixed dementia; MCI, mild cognitive impairment; MMQ, metamemory questionnaire for older adults; MMSE, Mini-mental state examination; N, sample size; PFT, phonemic fluency task; PMIT, picture memory impairment test; RBANS, assessment of neuropsychological status; RMBT, Rivermead behavioral memory test; so-tDCS, slow oscillatory tDCS; TMT, Trail making test; VAT, visual attention task; VD, Vascular dementia; VRT, visual recognition task; VWMT, verbal working memory task; RCT, randomized control trial; WAIS-III, Weschler abbreviated scale of intelligence; WRT, word recognition task. Values are means ± SD or as otherwise*.

### tDCS parameters

**Figure 2:**
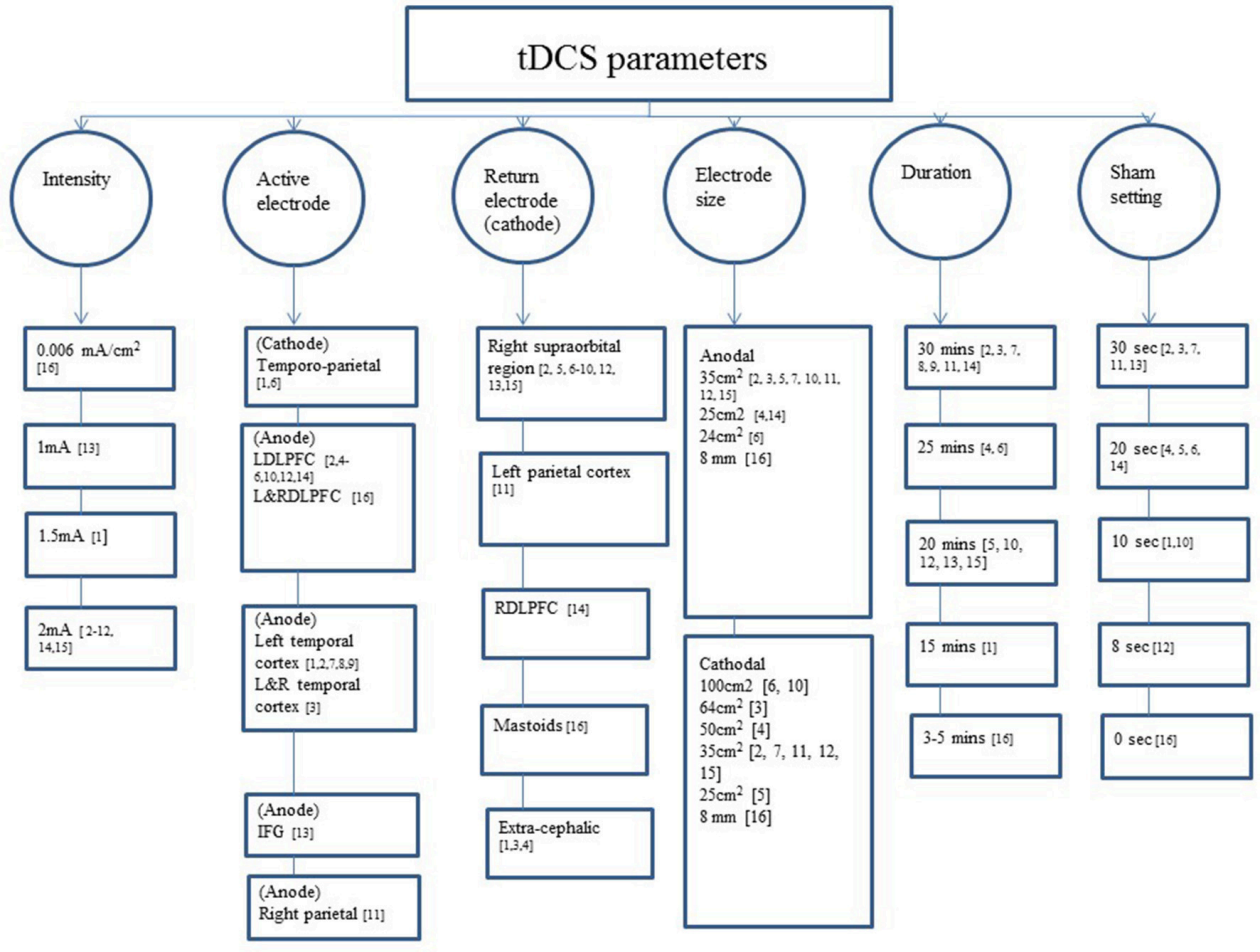
tDCS parameters used across the studies included. IFG: 1, (Ferrucci et al., 2008); 2, (Boggio et al., 2009); 3, (Boggio et al., 2012); 4, (Cotelli et al., 2014); 5, (Suemoto et al., 2014); 6, (Khedr et al., 2014); 7, (Bystad et al., 2016a); 8, (Bystad et al., 2016b); 9, (Bystad et al., 2017); 10, (Penolazzi et al., 2015); 11, (Costa et al., 2017); 12, (André et al., 2016); 13, (Meinzer et al., 2015); 14, (Yun et al., 2016); 15, (Murugaraja et al., 2017); 16, (Ladenbauer et al., 2017); IFG, inferior frontal gyrus; L/DLPFCT, left/right dorsolateral prefrontal cortex; L&R, left and right.

Two studies randomly assigned participants to anodal, cathodal, and sham groups (Ferrucci et al., 2008; Khedr et al., 2014). The majority of the studies involved anodal and sham groups (Boggio et al., 2009, 2012; Cotelli et al., 2014; Suemoto et al., 2014; Meinzer et al., 2015; Penolazzi et al., 2015; André et al., 2016; Bystad et al., 2016a; Yun et al., 2016; Costa et al., 2017; Ladenbauer et al., 2017; Murugaraja et al., 2017). In contrast, three studies focused on anodal stimulation lacking sham tDCS (Bystad et al., 2016b, 2017; Murugaraja et al., 2017). Regarding the dose, we found a high level of heterogeneity among experiments. Only four studies were single-session (Ferrucci et al., 2008; Boggio et al., 2009; Meinzer et al., 2015; Ladenbauer et al., 2017) whereas the number of sessions for the rest of studies ranged from 4 to 10 (Cotelli et al., 2014; Khedr et al., 2014; Suemoto et al., 2014; Penolazzi et al., 2015; André et al., 2016; Bystad et al., 2016a; Yun et al., 2016). Bystad carried out two case studies adopting unusual approaches, the first study with a daily dose of tDCS for a duration of 8 months (Bystad et al., 2017) and the second study using tDCS twice daily consecutively for 6 days (Bystad et al., 2016b). With respect to the electric fields, more homogeneous parameters were chosen among studies. The majority of the studies applied 2 mA of intensity and the targeted region for the active electrode was the DLFPC and the right supraorbital region for the cathode (Figure 2).

Six studies reported mild adverse reactions such as itchy and tingling sensations, redness in the area of electrode application, burning scalp, headache, dizziness, and pricking (Ferrucci et al., 2008; Khedr et al., 2014; Suemoto et al., 2014; Bystad et al., 2017; Ladenbauer et al., 2017; Murugaraja et al., 2017).

### Effectiveness of tDCS “alone”

Seven studies on the dementia population reported positive effects of anodal (Ferrucci et al., 2008; Boggio et al., 2012; Khedr et al., 2014; André et al., 2016; Bystad et al., 2016b, 2017) and cathodal tDCS (Khedr et al., 2014) on cognition. All these cognitive improvements were associated with memory and global cognition. All outcomes but two (Boggio et al., 2012; Bystad et al., 2017) were statistically significant. However, two of these studies failed to report positive effects in the attention domain (Ferrucci et al., 2008; Boggio et al., 2012). Two others did not report any positive effects of anodal tDCS on cognition (Suemoto et al., 2014; Bystad et al., 2016a).

Four studies (Boggio et al., 2012; Cotelli et al., 2014; Khedr et al., 2014; Bystad et al., 2016b) assessed the long-term effects of tDCS. Three of these reported significant changes: one showed that the improvement caused by anodal tDCS persisted 4 weeks after the end of stimulation (Boggio et al., 2012), another indicated that either anodal or cathodal tDCS improved mean MMSE score at 1- and 2-month follow-up (Khedr et al., 2014), and the third study revealed that 2 months after the end of the intervention, anodal tDCS was clinically significant (Bystad et al., 2016b).

Only two studies performed neuroimaging tests. In the first, an ERP experiment confirmed significant effects reducing P300 latency after both anodal and cathodal tDCS (Khedr et al., 2014). The second used EEG, although it did not prove changes from baseline (Bystad et al., 2016b).

Three studies evaluated the efficacy of anodal tDCS on PwMCI. Overall, anodal tDCS achieved significant improvement in memory (Yun et al., 2016; Murugaraja et al., 2017). Furthermore, two of these studies investigated the neural effects of anodal tDCS. Yun et al. (2016) utilized PET to demonstrate a significantly increased metabolism in cortical regions. In the same way, the work of Ladenbauer et al. (2017) made clear, through the use of concurrent EEG, that slow oscillatory tDCS significantly increased overall slow oscillations (SO) and spindle power (Ladenbauer et al., 2017).

### Effectiveness of tDCS combined with CT

Details and methods about the CT operated among studies are shown in Table 3. All the studies involving PwD showed significant benefits after receipt of anodal tDCS paired with a CT. Boggio et al. (2009)applied tDCS while participants completed cognitive assessments, enhancing memory in a visual recognition memory task, but there were no effects on attention. The work of Cotelli et al. (2014) combining memory training with tDCS and sham tDCS resulted in improved memory performance illustrated in a face-name association memory task, as compared to a group which received tDCS paired with motor training; this improvement persisted significantly after 12 weeks. However, it failed to produce significant effects on standardized cognitive tests. In one single-subject crossover study, the cognitive training associated with memory components was started right after the end of tDCS administration and the findings revealed a significant accuracy improvement in a verbal working memory task. In contrast, there is no indication of amelioration in other cognitive assessments (Penolazzi et al., 2015). Alternatively, one case study that focused on stimulating the production and comprehension of language through a combination of anodal tDCS and linguistic training found a significant effect in an auditory comprehension task (Costa et al., 2017).

The work of Meinzer et al. (2015) targeting PwMCI revealed that during exposure to anodal tDCS, participants performed significantly better in a semantic word-retrieval task than those who received sham tDCS, achieving the level of healthy elderly subjects. Furthermore, the application of anodal tDCS led to reduced task-related prefrontal hyperactivity shown by resting-state fMRI.

### Details of the CT

#### Study quality

The level of evidence of all the trials is displayed in Figure 1. Details can be found in Table 4. Most of the studies reported a risk of bias describing the method used to conceal the allocation sequence (Ferrucci et al., 2008; Boggio et al., 2012; Meinzer et al., 2015; André et al., 2016; Bystad et al., 2016a; Yun et al., 2016; Ladenbauer et al., 2017). The most common methodological limitation of these studies was the issue of the blinding of the personnel due to the nature of most tDCS devices.

**Table 4:** Methodological quality (Cochrane Risk of Bias Tool).

**Study**	**Random sequence generation**	**Allocation concealment**	**Blinding of participants and personnel**	**Blinding of outcome assessment**	**Incomplete outcome data**	**Selective reporting**	**Other bias**
Ferrucci et al., 2008	Unclear	High	High	Low	Low	Low	Low
Boggio et al., 2009	Unclear	Low	High	High	Low	Low	Low
Boggio et al., 2012	Unclear	High	Low	High	Low	Low	Low
Cotelli et al., 2014	Unclear	Unclear	High	Low	High	Low	Low
Suemoto et al., 2014	Low	Low	High	Low	Low	Low	Low
Khedr et al., 2014	Low	Low	Low	Low	Low	Low	Low
Bystad et al., 2016a	Low	High	Low	Low	Low	Low	Low
André et al., 2016	Unclear	High	High	High	Low	Low	Low
Meinzer et al., 2015	Unclear	High	High	Low	Low	Low	Low
Yun et al., 2016	Low	High	Low	Low	Low	Low	Low
Ladenbauer et al., 2017	Unclear	High	High	High	Low	Low	Low

#### Meta-analysis

**Figure 3:**
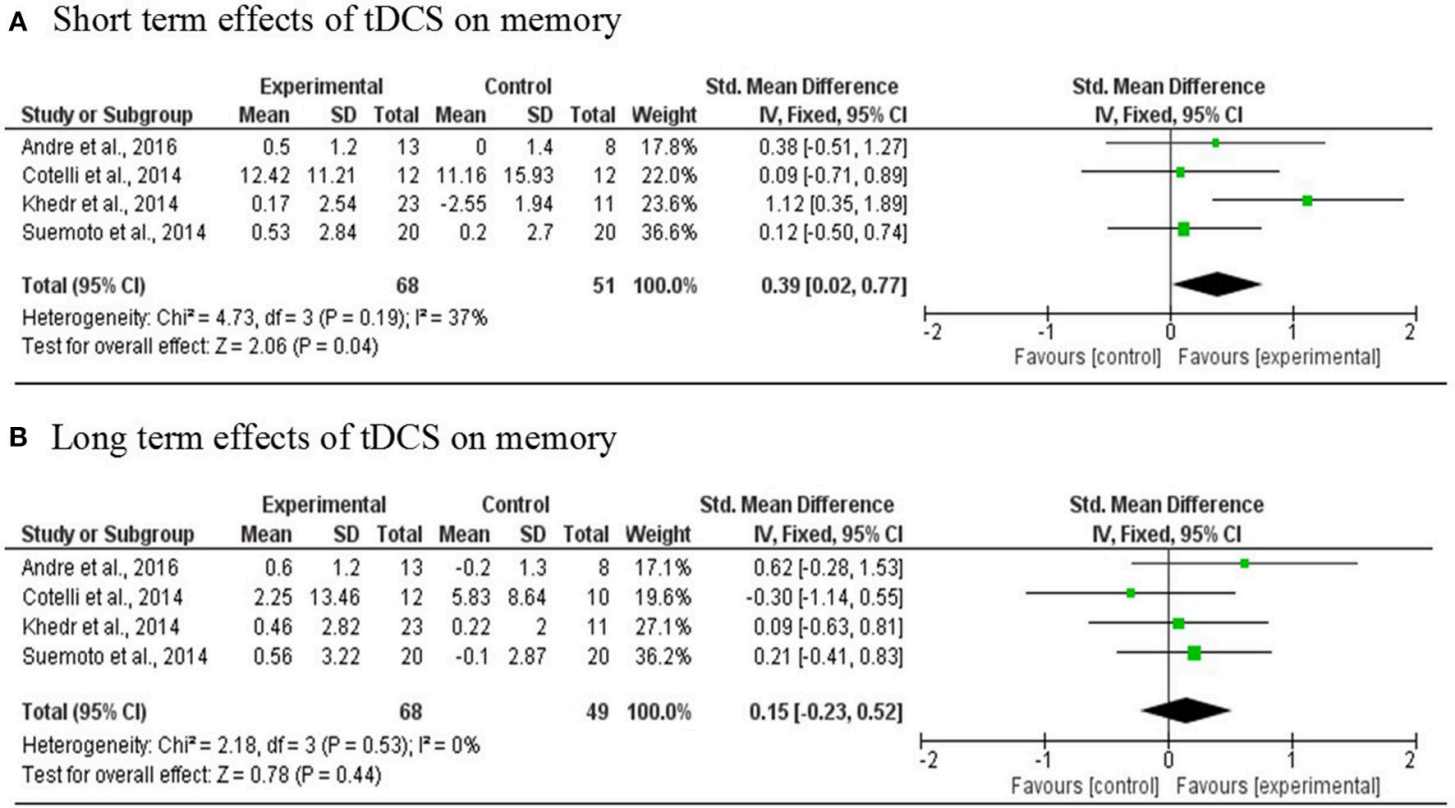
Meta-analyses forest plot. **(A)** Short term effects of tDCS on memory. Data derived from a fixed effect model. Each line represents an individual effect size of each study. The diamond at the bottom shows the standardized effect size (0.39). Relative weight for each trial is illustrated by the sized of the corresponding square. **(B)** Long term effects of tDCS on memory. Data derived from a fixed effect model. Each line represents an individual effect size of each study. The diamond at the bottom shows the standardized effect size (0.15). Relative weight for each trial is illustrated by the sized of the corresponding square.

Four studies (Cotelli et al., 2014; Khedr et al., 2014; Suemoto et al., 2014; André et al., 2016) involving 119 PwD in total were included in the meta-analysis. One RCT study was excluded because the region of stimulation was the temporal region (Bystad et al., 2016a). The results revealed a statistically significant mean effect size of 0.39 [95% CI, 0.02, 0.74] (*p* = 0.04) that favored real tDCS over sham stimulation for immediate effects. There was no evidence of heterogeneity across studies (Q = 4.73, *I*^2^ = 37%, *p* = 0.19). An overall small non-significant effect of 0.15 [95% CI, −0.023, 0.52] (*p* = 0.44) was noted in long-term effects of tDCS in comparison with sham tDCS. Heterogeneity was not found (Q = 2.18, *I*^2^ = 0%, *p* = 0.53; Figure 3).

## Discussion

All the 11 articles (RCTs) whose evidence was ranked as level 1b presented a commendable methodological quality with a general presence of low risk of bias. From the MMSE admission scores in the AD studies that ranged from 15 to 24.5 and MCI studies from 26.75 to 28.3, we noticed that the effects of tDCS benefits on cognition were significantly better for patients with mild to moderate cognitive decline.

When comparing the effectiveness of tDCS, in single and multisession interventions, positive changes occurred in both behavioral and neural systems. In this systematic review, we aimed to reveal robust interventions by identifying similar elements across studies. One main concern when designing interventions in NIBS is the treatment duration in multisession trials. There is similarity in terms of the number of sessions across the selected studies: four to ten sessions, staggered over 1–2 weeks. These short interventions can provide valuable data that allow tDCS to be proposed as a potential option in CR. However, the benefit is rather short-term with a medium effect size of 0.39. This also contrasts with other long intervention frameworks for clinical use in which more time is needed to evaluate whether the changes have a real benefit in reversible conditions such as MCI (Portet et al., 2006) or have an impact in long-term neurodegenerative processes such as dementia. For example, an alternative was proposed by Bystad et al. (2017) that adopted an 8-month protocol of daily tDCS use in a person with AD to stabilize cognitive decline. The long-term outcome probably requires prolonged periods of intervention.

Although six studies reported side effects (Ferrucci et al., 2008; Khedr et al., 2014; Suemoto et al., 2014; Bystad et al., 2017; Ladenbauer et al., 2017; Murugaraja et al., 2017), all participants tolerated the therapies well and the sensations experienced were mild. This suggests that the parameters employed are sufficiently safe (up to 30 min, 2 mA). Another concern is that the range of the parameters for intensity and duration stimulation and the size of the electrodes were highly diverse, making it difficult to draw conclusions in order to select a specific protocol for future research.

Another view is that when selecting a region of interest for stimulation, most of the studies targeted the temporal regions (Ferrucci et al., 2008; Boggio et al., 2012; Bystad et al., 2016a,b, 2017), for the role this area plays in certain memory processes (Brown et al., 1987; Kaye et al., 1997) as well as language (Nguyen et al., 2018). Another common region of interest is the DLPFC because of its importance in high-order cognitive mechanisms (Tremblay et al., 2014). Language-oriented work has targeted the inferior frontal gyrus and DLPFC as well, successfully achieving better performance in semantic word retrieval (Meinzer et al., 2015) and comprehension of language (Costa et al., 2017). In the same way, studies that applied tDCS combined with CT operated a CT related with a cognitive domain associated with the brain area targeted by tDCS. Although this approach is reasonable and consistent, the studies failed to assess if other cognitive domains associated with other brain regions were affected. Due to the lack of focality of tDCS and the variability of the current flow direction, there is a possibility that other neural networks, not directly targeted by tDCS, could have been affected (Woods et al., 2016).

Three studies used an extracephalic cathodal montage (Ferrucci et al., 2008; Boggio et al., 2012; Cotelli et al., 2014) but the majority of the studies selected a cephalic montage by placing the cathode on the supraorbital region (Fp2) (Boggio et al., 2009; Khedr et al., 2014; Suemoto et al., 2014; Meinzer et al., 2015; Penolazzi et al., 2015; André et al., 2016; Bystad et al., 2016a,b, 2017; Costa et al., 2017; Murugaraja et al., 2017).

Overall, these studies have selected predominantly global cognition and memory domain as experimental evaluators. Despite the fact that these constructs are similar in nature, there is great variability in terms of assessment and CT chosen. All the studies but two (Suemoto et al., 2014; Bystad et al., 2016a) report positive effects of the application of tDCS. Against this trend, among the other articles, we must emphasize that only six studies translated these improvements into standardized cognitive assessments (Ferrucci et al., 2008; Khedr et al., 2014; André et al., 2016; Bystad et al., 2016b; Yun et al., 2016; Ladenbauer et al., 2017) while other studies reporting improvements in non-standardized CT to prove the effects of tDCS. Yet it must be acknowledged that certain cognitive functions are mediated by networks of various brain sites and might be difficult to be influenced by targeting only a subset of their brain regions (Reinhart et al., 2017), besides the short length of the intervention might have contributed to these changes being insufficient to translate into standardized test results.

It is hypothesized that targeting a neural circuit with tDCS paired with a CT may produce stronger therapeutic effects than stimulating the same brain area without cognitive stimuli (Birba et al., 2017; Cruz Gonzalez et al., 2018). The evidence on whether using tDCS alone or in combination with other CT yields identical results and seems to be inconclusive in both PwD or PwMCI. Recently, a single-subject design study using cognitive stimulation practice across sessions in combination with simultaneous anodal tDCS showed significantly stronger effects on planning ability, processing speed, and attention of cognitive stimulation practice than both sham tDCS and the application of cognitive stimulation practice alone in PwMCI (Cruz Gonzalez et al., 2018). This finding prompts the plausible speculation that tDCS, combined with cognitive training, might have synergic effects. A recent review of CR or cognitive training interventions with control conditions for PwD shows that RCTs on the effect of cognitive training on PwD are limited and there is no indication of any significant benefits from cognitive training (Bahar-Fuchs et al., 2013). Following this line of thought, future studies would carry more weight if they considered combining both interventions in comparison with control groups receiving tDCS or cognitive training alone, and would report not just benefits in the trained CT but also generalization to the trained cognitive domains and daily functioning.

Only five studies reported the use of brain imaging as an outcome demonstrating the neuromodulatory effects of tDCS (Khedr et al., 2014; Meinzer et al., 2015; Bystad et al., 2016b; Yun et al., 2016; Ladenbauer et al., 2017). In the absence of imaging techniques, we can only speculate on the results of behavioral tests without examining the underlying neural mechanism of tDCS in MCI or dementia.

This is the first meta-analysis to explore the short- and long-term effects of tDCS in the memory domain, targeting the DLPFC in PwD. We have found evidence that tDCS has a significant immediate effect but that it is not significantly sustained with the passage of time. We suggest that future research address the need to evaluate the long-lasting effects of tDCS on the cognitive domain, implementing both behavioral and imaging follow-up evaluations.

This study has several limitations. For instance, although the pooled outcomes for meta-analysis were all memory-based, the selected studies used different tests. In addition, only four studies could be included, this might contribute to making the meta-analyses somewhat underpowered, thus the findings should be interpreted with cautions. Another striking example is the AD stage, which varied among the studies. Moreover, we have not included the most recent work published since November 2017 (Cruz Gonzalez et al., 2018), because of the time eligibility criteria. This systematic review included all tDCS trials carried out in dementia and MCI populations, and subsequently reported a few papers that did not use a comparison group (sham tDCS), which weakens the conclusions somewhat.

## Conclusion

Our meta-analysis suggests that there is modest evidence supporting tDCS on the DLPFC ameliorates memory in PwD, however, the benefits are not long-term. Our review shows that tDCS alone seems to have a positive effect on cognition particularly for memory and language in PwD, with mild to moderate cognitive decline, and MCI. Whether tDCS might produce better outcomes on PwMCI and PwD in coupling with another CT than when administered alone remains unclear.

Although all these findings are promising, the administration of tDCS might not yet be a valid option for clinical intervention for dementia or MCI. Some of the results come from non-RCT studies, and the heterogeneity of the clinical trials does not allow one to define a clear protocol with optimal parameters. Furthermore, the interventions were too short to determine the real effects on cognitive functions and none of the studies assessed the impact of treatments on everyday cognition in daily functioning, which is an essential domain to be considered due to the functional consequences of dementia. We recommend that future studies include prolonged periods of intervention, neuroimaging techniques, and consider more robust, standardized methodology of tDCS in order to establish whether tDCS can serve as an evidence-based clinical intervention for PwMCI and PwD.

## Author contributions

PC and KF designed the study, collected the data, conducted the statistical analysis, and wrote the manuscript. RC supervised the statistical analysis. K-HT and LL provided advice writing the manuscript. TB supervised the design and provided guidance.

### Conflict of interest statement

The authors declare that the research was conducted in the absence of any commercial or financial relationships that could be construed as a potential conflict of interest.

## Footnotes

^1^https://www.cebm.net/2009/06/oxford-centre-evidence-based-medicine-levels-evidence-march-2009 (Accessed March 29, 2018).
